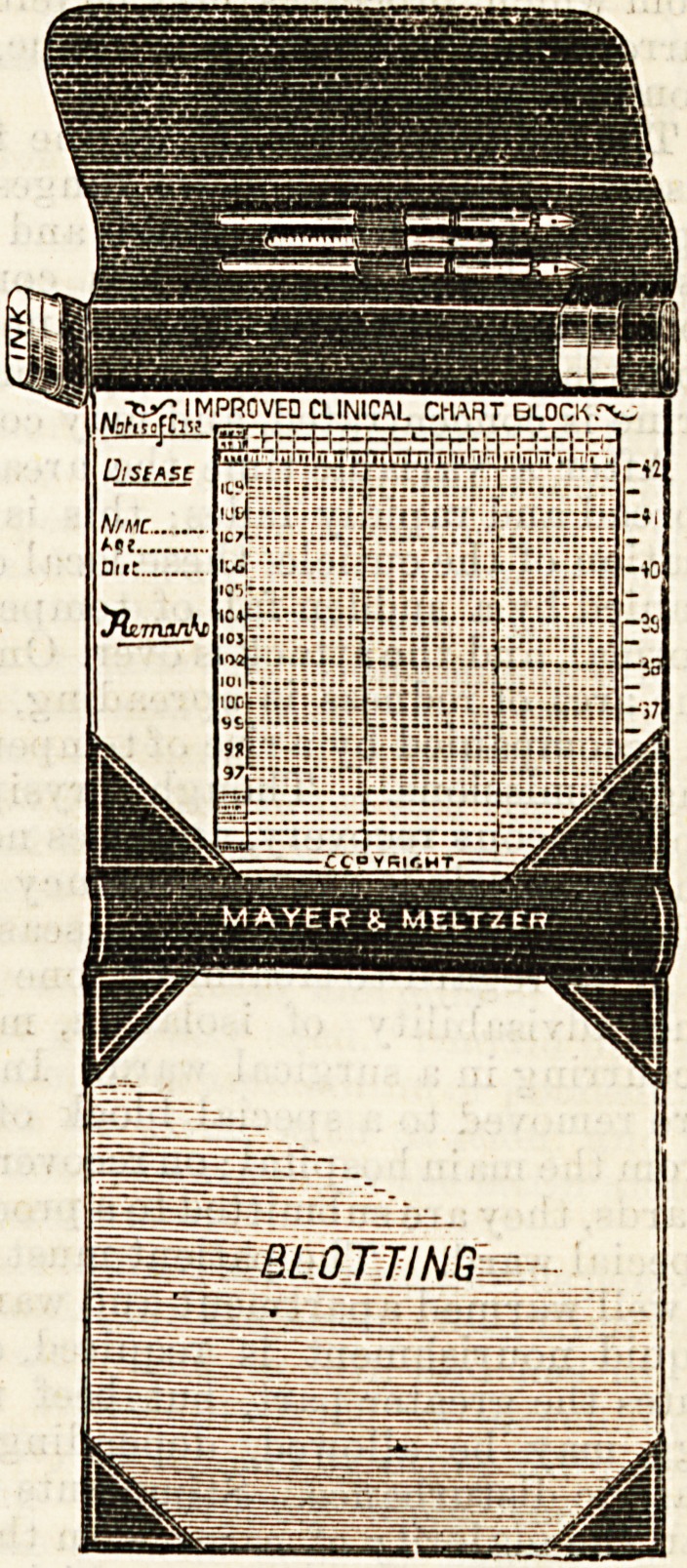# New Appliances and Things Medical

**Published:** 1893-11-18

**Authors:** 


### NEW APPLIANCES AND THINGS MEDICAL.

### IMPROVED CLINICAL CHART BLOCK AND CASE.

The above illustration shows a convenient and portable form of temperature chart block and case. It was suggested by an experienced nurse, and has been found most useful in practice. It combines a handy case for the registered chart block (which can be had separately), ink bottles, pens, and clinical thermometer. The advantage of the chart block (from which the sheets can be separated when filled) is obvious, the single sheets being usually mislaid before completion. A pocket is made under the blotting pad to contain the used charts. This block is one of the handiest things we have seen for a long time, and, together with its case, forms an item of a general practitioner's, student's, and nurse's requisites, which neither of them, especially if engaged in private work, can afford to be with out. It will pack up very easily into a travelling bag, and ought to be especially popular just now when the time for Christmas presents is near at hand. It can be used as a writing case as well as for temperature charts. It has been made by Messrs. Mayer and Meltzer, of Great Portland Street, London.

## Figures and Tables

**Figure f1:**